# Challenges in validation of combination treatment strategies for CRC using patient-derived organoids

**DOI:** 10.1186/s13046-024-03173-x

**Published:** 2024-09-11

**Authors:** Valentin Benboubker, George M. Ramzy, Sacha Jacobs, Patrycja Nowak-Sliwinska

**Affiliations:** 1 Molecular Pharmacology Group, School of Pharmaceutical Sciences, University of Geneva, 1 Rue Michel-Servet, Geneva, 4 1211 Switzerland; 2 Institute of Pharmaceutical Sciences of Western Switzerland, University of Geneva, Geneva, 1211 Switzerland; 3 Translational Research Center in Oncohaematology, Geneva, 1211 Switzerland; 4 Department of Cell Physiology and Metabolism, Faculty of Medicine, University of Geneva, Geneva, 1211 Switzerland

**Keywords:** Combinatory treatment, Immunotherapy, Intestinal organoids, Intestinal tumor organoids, Precision medicine, Synergy evaluation, Tumor microenvironment

## Abstract

Patient-derived organoids (PDOs) established from tissues from various tumor types gave the foundation of ex vivo models to screen and/or validate the activity of many cancer drug candidates. Due to their phenotypic and genotypic similarity to the tumor of which they were derived, PDOs offer results that effectively complement those obtained from more complex models. Yet, their potential for predicting sensitivity to combination therapy remains underexplored. In this review, we discuss the use of PDOs in both validation and optimization of multi-drug combinations for personalized treatment strategies in CRC. Moreover, we present recent advancements in enriching PDOs with diverse cell types, enhancing their ability to mimic the complexity of in vivo environments. Finally, we debate how such sophisticated models are narrowing the gap in personalized medicine, particularly through immunotherapy strategies and discuss the challenges and future direction in this promising field.

## Introduction

The widespread discussion on the ethics of animal experimentation emphasizes the necessity for the development of alternative in vitro models to advocate for the “3Rs initiative” that represent *reduction*, *refinement*, *and replacement* of laboratory animals [1]. Legislation signed in late December 2022, indicates a significant shift in drug safety regulation, as it eliminates the requirement for animal testing to obtain U.S. Food and Drug Administration approval for new medicines [2]. This marks a radical change from over 80 years of reliance on animal experimentation in the realm of drug safety. While drug discovery cannot rely solely on in vitro models, the establishment of solid pre-clinical ex vivo platforms using patient-derived material, can yield valuable outcomes that, in turn, contribute to minimizing the need for additional in vivo experiments and facilitating clinical transition.

For many years, two-dimensional (2D) cell cultures and in vivo tumor models have been the standard for drug discovery and cancer research [3]. Multiple research groups have elegantly reviewed the limitations of 2D models in current drug development while introducing new state of the art in vitro and ex vivo models [3–5]. In 2011, Sato et *al*. established for the first-time colorectal cancer organoids from freshly isolated patient-derived tissue [6]. Since then, the establishment of organoids as pre-clinical model with patient-specific signatures allowed bridging the gap between 2D and more complex models of colorectal cancer (CRC) [7]. Organoids are self-organising 3D tissues, which mimic essential functional, structural, and biological characteristics, as well as histopathological and genomic features of the tissue they derive from [7]. They can be cultivated from tissue-derived cells, induced pluripotent stem cells or from surgically removed patient samples [8–10]. The cultivation of PDOs requires an interdisciplinary network between fundamental researchers and clinicians, including surgeons, oncologists and clinical pathologists. Close collaboration between these groups within the team is essential to secure optimal conditions from tissue resection to model establishment [4]. In experimental precision oncology, such models serve for various applications, including drug screening, optimization of immunotherapies, validation of combination treatment, design of personalized drug regimen, mechanistical evaluation, and biomarker identification. Consequently, organoids have emerged as relatively inexpensive and representative platforms for modelling cancer heterogeneity and interactions with the tumor microenvironment (TME) in vitro [11].

CRC is a heterogenous tumor commonly defined by four Consensus Molecular Subtypes (CMS). Each subtype harbors a different immune landscape and tumor-microenvironment [12]. For example, CMS1 is characterized by MSI high tumor phenotype and is associated with a high T cell infiltration and activation. This CMS1 group is the most likely to respond to immunotherapy. On the other hand, the CMS2 and CMS3 are poorly immunogenic, characterized with a low immune cell infiltration. Finally, the CMS4 is strongly enriched with immunosuppressive cell populations (such as regulatory T cells (Treg) and M2-like macrophages) and stromal cells. The latter three CMS subtypes represent microsatellite stable (MSS) tumor phenotype and are less likely to respond to immunotherapy, thus a better understanding of those CMS subtypes will guide future drug selection and combination [12–15].

It was demonstrated that tumor organoids have a broad conservation of the phenotype of tumor cells [8, 10, 16, 17]. In addition, the cytokeratin 20 positive and cytokeratin 7 negative (CK20^+^/CK7^−^) immunophenotype, highly characteristic of CRC tumors, was observed in the majority of PDOs [8]. Furthermore, the expression of Ki67, CDX2, a homeobox gene that marks intestinal differentiation,

β-catenin, cytokeratins (CK), and especially CK20, all potential markers for the clinical diagnosis of CRC, was retained in organoids compared to the primary tumor Sect. [16]. Additionally, expression levels of mismatch repair (MMR)-related proteins, including MLH1, MSH6, MSH2, and PMS2, were detected in the PDOs [16]. With whole exome sequencing, researchers demonstrated a similarity in 96% mutations in key driver genes comparing PDOs and primary tumors [10, 17]. However, certain samples exhibited new somatic variants that were not detected in the primary tumor [8]. Overall, in most of the cases the PDOs generated resembled the patients’ primary tumor. On average, there is 76% accuracy in organoids predicting patient response, with a sensitivity of 0.79 and a specificity of 0.75 [18], making PDOs suitable to guide the selection of an effective therapeutic approach, personalized to each patient [9, 10, 17, 19–21]. Similarly, in case of organoids isolated from the liver, where CRC metastases are often located (mCRC), PDOs exhibited comparable responses to patient outcomes, underlining their clinical relevance [22]. Additionally, using PDOs cultivated from refractory mCRC patients, who underwent two standard of care treatment protocols, next-generation sequencing and subsequent drug screen analyses, yielded clinical results for four patients. Within three weeks, these patients received their recommended therapies, resulting in a minimum of 5 months stable disease [23]. However, not all significant responses in PDOs can be translated in effective clinical outcomes, indicating the need for further refinement in PDO-guided therapies [24]. Thus, while PDOs hold potential in personalized CRC treatment selection, continued research is essential to optimize their clinical utility and overcome existing limitations.

It is currently well acknowledged in the field that combinatory treatment holds better potential of cure over monotherapy interventions [25]. For cancer treatment, a multifactorial disease, such single target agents used at high doses have shown limited efficacy due to induction of resistance and relatively strong side effects [25, 26]. This paved the way for poly-pharmacology as a more potent therapeutic tool [27, 28], notably for complex diseases that fail to respond to monotherapy treatment strategies [29, 30]. Combinatory pharmacological treatment is defined as the use of pharmaceutical agents acting on multiple molecular targets or multiple biochemical pathways. This can be achieved by combining drugs, targeting different signaling pathways or by designing a drug that acts on multiple targets with various affinities. Nowadays, it is clinically achieved by the use of chemotherapy based combination, FOLFOXIRI (FOL: folinic acid; F: 5-FU; OX: oxaliplatin; IRI: irinotecan), as main or adjuvant treatment modality in different stages of CRC [31].

This attractive avenue as a novel cancer treatment strategy is still in its infancy, specifically for CRC, as our knowledge on the complex drug-drug interactions in the cancer environment is still not fully elucidated. Many challenges remain on how to (i) select the drug candidates and their dose, (ii) which parameter to evaluate the efficacy and toxicity of such combinations, (iii) which relevant pre-clinical cellular model to use for a facilitated clinical translation or how to (iv) evaluate drug-drug interactions [32]. Current advances in ‘omics’ technologies, together with *in silico* modelling and whole genome sequencing, have greatly improved the workflow and have contributed to drug (combination) development discovery, yet the validation in organoid models remains rather rare.

In this review, we focus upon PDOs application for the validation and optimization of multi-drug combinations as part of personalized treatment strategies for CRC. We discuss recent efforts to enrich organoid models, contributing to mimic the complexity of the in vivo environment. We address how such advanced models are utilized to bridge the gap for better tailored clinical translation using immunotherapeutic strategies, along with consequent challenges and potential future directions.

### Combination of targeted therapies in CRC treatment-naïve and treatment-resistant organoids

Despite the strong potential attributed to PDOs as a pre-clinical model in translational oncology and therapy development against cancer, dedicated drug-combination screens for personalized treatment strategies for CRC using PDOs are scarcely reported. When it comes to combinations of targeted therapies, organoids have been employed in different modalities. Either to validate already established drug combinations, or to directly screen for new combinatorial treatments.

#### Treatment-naïve tissues

Multiple studies have highlighted the strong therapeutic potential of the combination of MEK and PI3KCA inhibitors [33, 34], using CRC 3D models [30, 35], however, very few validated this combination strategy directly in CRC PDOs. Atanasova et *al*. elegantly presented the difference of response to combination therapy in CRC according to a model used [36]. In their study, no difference in efficacy was reported between the triple-drug combination consisting of Torin 1 (mTOR1/2 inhibitor), MK2206 (AKT inhibitor) and selumetinib (MEK1/2 inhibitor), when compared to the efficacy of investigational Gedatolisib (pan-class I isoform PI3K and mTOR inhibitor) combined with selumetinib, in 2D and homogeneous 3D spheroids of DLD1 colon cancer cells. However, difference in activity was seen only in PDOs derived from both primary tumor and liver metastases CRC patients, harboring mutations in *PI3K* and/or *RAS-RAF-MAPK* pathways.

**Fig. 1:**
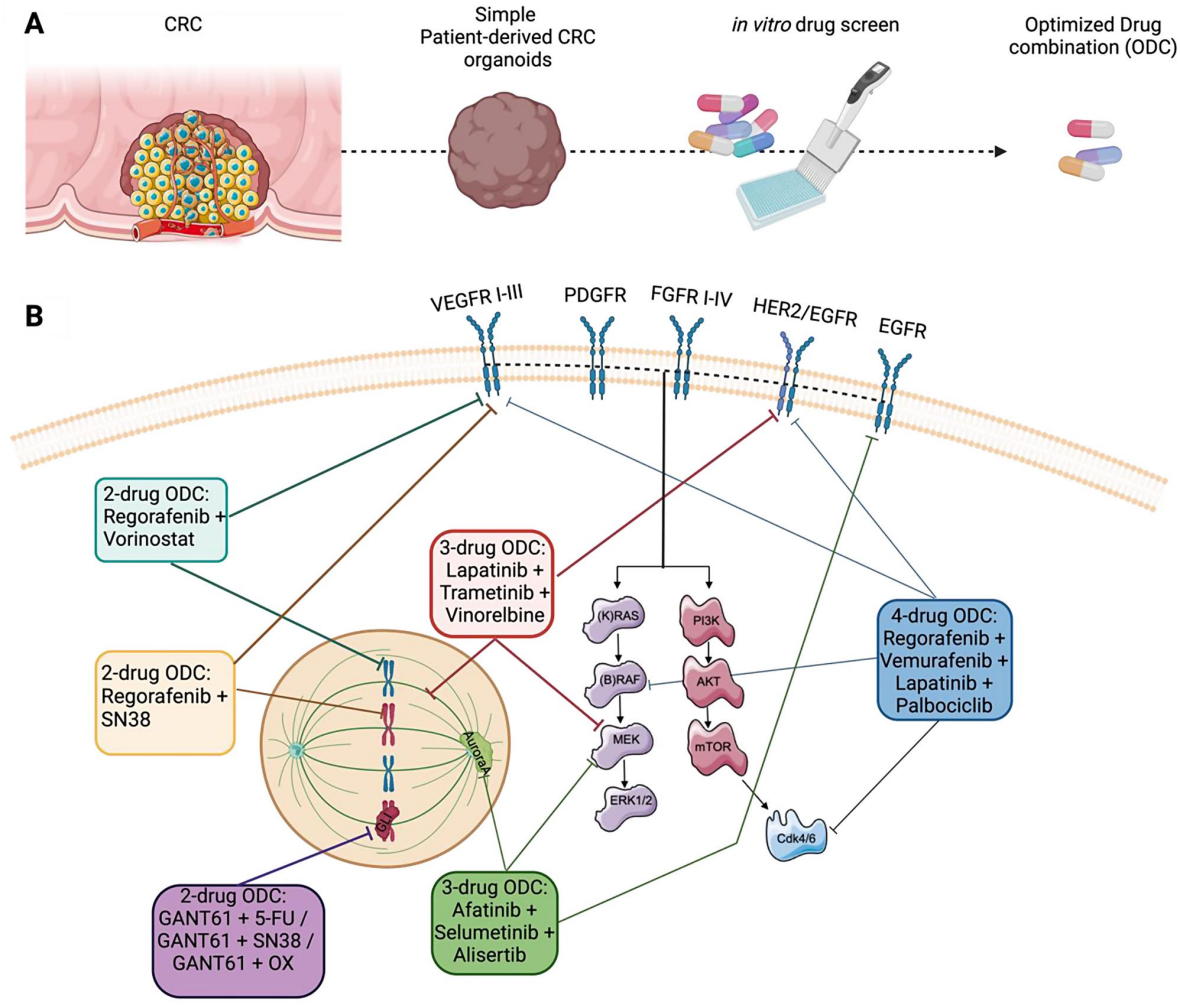
Organoids formation and signaling pathways of optimized drug combinations (ODCs). (**A**) Schematic representation of different steps in an in vitro drug screening process. (**B**) Different combinations of targeted therapies optimized using CRC PDOs and their known targeted signaling pathways. Created with BioRender

Another main strategy in leveraging the full potential of PDOs, consists in directly using the organoids technology in optimizing combinatory treatment strategies for CRC patients. In a recent study conducted by our research team, Ramzy et *al*. established an innovative platform for the rapid optimization of drug combination (ODC) using individually grown PDOs tailored specifically to individual patients [19]. To do so, the authors had to preliminarily optimize a culture seeding method that allows the organoids technology to be adapted to a high throughput drug screen (Fig. 1A). Their organoid culture procedure allowed to reproducibly culture single organoids per well at a standardized size of 350–450 μm. This proprietary phenotypically-driven platform called Therapeutically-Guided Multidrug Optimization (TGMO) [19, 35, 37], was applied to systematically screen combinations of tyrosine kinase inhibitors (TKIs) on PDOs obtained from individuals with primary and metastatic CRC, in a clinically relevant timeframe. Specifically, the ODC comprising of four drugs (Fig. 1B), i.e. regorafenib, vemurafenib, palbociclib, and lapatinib applied at low doses showcased a substantial inhibition of cell viability, achieving up to 88% reduction, in PDOs derived from a patient with CRC liver metastases (stage IV), characterized as CMS4/ CRC intrinsic subtype A (CRIS-A). In addition, the activity of the ODC showed a significant therapeutic window when evaluated on non-cancerous colon model. Moreover, the efficacy of the patient-specific ODCs was found to significantly surpass that of FOLFOXIRI, at clinically used doses. These findings underscored the potential of the TGMO platform in tailoring cancer treatment strategies by leveraging ex vivo patient material, in a clinically relevant time frame of 2–3 weeks [19].

Using as similar design of experiment as described previously in the study of Ramzy et *al*., Thng et *al*. applied a phenotypic approach, i.e. Quadratic Phenotypic Optimization Platform, to screen for synergistic two-drug combinations. It was done using PDOs from primary and metastasis lesions [38]. Interestingly, for two of the three pairs of matched PDOs, the authors found similar sensitivities in both primary and corresponding metastasis-based PDOs to the patient-specific synergistic two-drug combinations (Fig. 1B), i.e. regorafenib + SN38 and regorafenib + vorinostat (HDAC inhibitor [39]), respectively. However, for the third patient, different drug combinations were optimized showing different efficacy between the metastases and the primary PDO. The authors limited their screen on drug pairs, highlighting the difficulty for more than 2 drugs to be administered systemically.

Mertens et *al*. introduced a phenotypically-driven approach using a microscopy-based strategy, to screen for a triple-drug combination that allows to stabilize the cytotoxic effect in advanced stage of CRC [40]. Their drug-repurposing screen yielded 34 positive hits out of 414 clinically approved drug candidates tested, in monotherapies or drug-pairs, on a panel of 36 KRAS-mutant CRC PDOs. By complementing their metabolic inhibition assay with microscopy, they were able to identify the microtubule-targeting agents, more specifically Vinorelbine^®^, as the most potent class of compounds to result in a switch from a drug-induced cytostatic phenotype, upon treatment with lapatinib (a dual HER2/EGFR inhibitor [41]), trametinib (MEK1/2 inhibitor [42]) at clinically used doses adapted to a patient clinical maximum plasma concentration (C_max_), to a drug-induced cytotoxic phenotype. The authors tested multiple combinations of Pan-HER/MEK used in trials (i.e. afatinib + selumetinib) and observed a similar effect. Even though synergy was higher for the triple-drug combination of lapatininb + trametinib + Vinorelbine^®^, the observed anti-tumor effects remained unchanged when the dual TKIs were replaced by other mechanistically-identical drugs (afatinib + selumetinib), provided that downstream MAPK pathway signaling was effectively inhibited (Fig. 1B). Their findings were further validated in vivo, by reproducing the clinical exposure in tumor mouse models, where 62% reduction in tumor growth was observed for the combination of lapatininb + trametinib + Vinorelbine^®^ compared to the sham-treated mice [40].

#### Chemo-resistant tissues

Described above dual inhibition using a combination of pan-HER2 and MEK1/2 inhibitors in addition to a third agent, has been also shown to be effective in CRC PDOs resistant to chemotherapy. Even though current treatment modalities for CRC, including chemo-/radio-therapy, targeted agents and immunotherapy have highly improved CRC patient survival, some patients have inherent genetic mutations that result in resistance to treatment, or lose sensitivity to treatment over time. These intrinsic or acquired resistance are mainly due to changes in tumor-drug metabolism, cellular transportation or target, as reviewed by others [43–45]. In a previous study, we have generated *in vitro and ex vivo* CRC cell models resistant to clinically used first line chemotherapy, FOLFOXIRI, concomitantly administered to the cells [19, 46, 47]. We highlighted the strong potential of ODCs in overcoming chemotherapy resistance in CRC, where different combinations of four TKIs inhibited up to 90% of viability in chemotherapy-resistant cells. However, this was shown in FOLFOXIRI-resistant cells cultured in 2D, and very limited number of studies have been carried out to optimize drug combinations on chemo-resistant organoids.

Boos et *al*. exposed KRAS and BRAF wild-type PDOs from three patients (one primary CRC metastatic tumor and two CRC liver metastases) to a chronic combination treatment of FOLFIRI + cetuximab, administered concomitantly, to establish ex vivo chemoresistance within a variable patient-dependent period from 4 to 6 months [48]. Interestingly, no mutational changes in KRAS were observed in all three PDOs after long-term exposure to the combination treatment. Therefore, the authors engineered a KRAS^G12D^ mutation using CRISPR/Cas9. This reduced the effect of cetuximab or a dual inhibition using afatinib + selumetinib in all PDOs tested. To overcome such resistance, the authors shed a light on Aurora A kinase as a potential target. The triple-therapy with afatinib + selumetinib + alisertib (Aurora A inhibitor) [49], illustrated in Fig. 1B, showed a significant reduction in cell viability of all PDOs that were EGFR-therapy resistant, through a concomitant cell cycle arrest in G2/M phase and the dual inhibition of EGFR pathway.

Usui et *al*. introduced a new strategy to overcome resistance to conventional chemotherapy in CRC. The authors underlined the strong potential of stem cell signal inhibitors in overcoming such resistance. They used their air-liquid interface (ALI) model [50], where patient-derived tissue, embedded in collagen layer submerged in intestinal stem cell media, formed ALI organoids over time recapitulating the intestinal epithelial and mesenchymal structure as the primary tumors. Their results highlighted that the combination of a Hedgehog signal inhibitors (AY9944 or GANT61) with 5-FU, irinotecan or oxaliplatin (Fig. 1B) significantly decreased the tumor organoids cell viability when compared to the activity of each chemotherapy alone. They further confirmed their results through protein levels expression, where the treatment of ALI organoids with AY9944 or GANT61 resulted in a downregulation of stem cell markers (c-MYC, Nanog and CD44) expression through downregulation of GLI-1 protein, known to be overexpressed in CRC chemo-resistant cell lines [50].

Even though the above-mentioned studies introduce novel approaches to optimize multi-drug combinations for CRC patients, the PDOs are still far from being ideal models as the immune, vascular and stromal compartments are not present. To leverage the full potential of drug combinations, more complex models need to be developed. Below we refer to studies where attempts to enrich the microenvironment of organoids are discussed.

### Microenvironment enrichments of PDOs

While PDOs maintain the tumor characteristics of a patient and can be predictive of their response to different treatments, as a preclinical model they have multiple limitations. This is mainly translated by the lack of certain actors present in TME, e.g. immune cells, endothelial cells and fibroblasts. While some PDOs from different cancer types preserved integrated components of the immune system [44], the majority of the PDOs do not contain immune cells [51].

#### Enrichment with immune cells

**Fig. 2:**
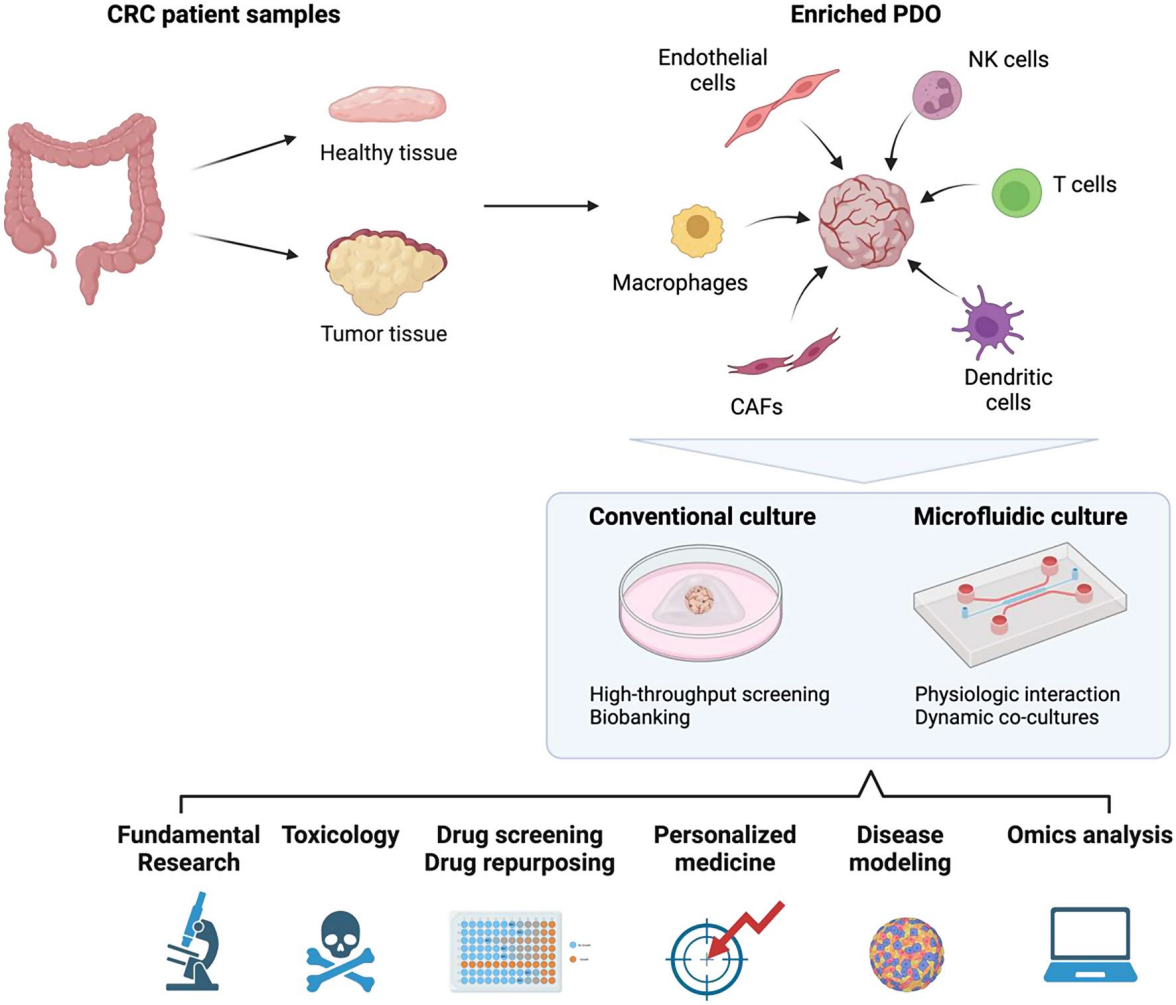
Schematic overview of various CRC organoid-based enriched models and their applications. Healthy tissue is used alongside tumor tissue to assess the toxicity of the drugs under study. Created with BioRender

Therefore, different studies reported complex co-culture models of CRC PDOs with the addition of different types of immune cells as schematically represented in Fig. 2. This, in turn, provides an opportunity to explore response and resistance mechanisms to both immune- and targeted- combination therapies [52]. *For instance*, with the addition of IL-2, integrated immune components in PDOs can be retained for up to 7 days [38]. However, since only a limited number of immune cells are preserved, an additional number of immune cells of different subtypes need to be present to more accurately represent the TME. Fang et* al*. cultured CRC PDOs with patient-derived CD8^+^ T cells together with macrophages isolated from the peripheral blood mononuclear cells (PBMCs) (Fig. 2). The latter was activated by the addition of macrophage colony stimulating factor (M-CSF) for 7 days before co-culturing them with the CRC organoids [53]. The levels of SIRT1 in CRC was shown to influence the TME and cancer progression. CRC samples with high expression of the protein, had decreased levels of CD8 + T cells, while the percentage of tumor-associated macrophages (TAM) was increased, inhibiting tumor proliferation and the anti-tumor activity of CD8 + T cells [53]. This study using organoids co-culture reflects the crosstalk complexity that can be observed in patient samples with the advantage of offering a real-time follow-up setting.

Other research groups reported the addition of dendritic cells (DCs) to the PDOs culture. DC are required for the detection, processing, and presentation of tumor antigens as well as the activation of antigen-specific T cells to orchestrate an effective antitumor response. Subtil et *al.* successfully incorporated monocyte-derived dendritic cells (MoDCs) differentiated with granulocyte-macrophage colony-stimulating factor (GM-CSF) and IL-4 for 5 days, from healthy donors into a 3D collagen matrix PDOs co-culture system. In the presence of PDOs, reduced expression of CD86 was observed, hinting towards a tumor-induced immunosuppressive effect. Immature DCs (iDCs) were activated with IL-6, IL-1b, TNF-a and PGE2, for 24 h before harvesting. In co-cultures of PDOs with iDCs or mature DCs (mDCs), iDCs were being significantly closer to the tumor and demonstrated higher infiltration compared to the mDCs [54]. Due to the fact that the iDCs were not matured, they had ability to induce T cells differentiation into regulatory T cells, which was associated with pro-tumorigenic effects [55]. Furthermore, the co-culture of conventional DC type 2 (cDC2) with PDOs (Fig. 2) resulted in an infiltration of the cells into the PDOs and the activation of T cells in the co-culture. The cytokine production and secretions by the PDOs (like PGE2 and IL-6) was shown to influence the conversion of cDC2 to DC3-like cells which has been linked to impairments in T cell proliferation and activation thus inducing an immunosuppressive environment [56]. In this context, assessing the immune cells phenotypes in co-culture is also important to understand what drive the pro or anti-tumoral environment that probably impact as well the drug response.

Moreover, Natural Killer (NK) cells gained significant attention recently [57, 58], specifically in CRC, where tumor-infiltrating NK cells and their cooperation with T cells [59, 60] has been linked to a good prognosis [61, 62], highlighting their important role in controlling tumor growth [63] and overcoming resistance to chemo- and T cell-based immune-therapy [64]. Lanuza et *al*. developed a 3D co-cultured CRC model with allogenic activated NK cells (Fig. 2), enriched with CD56 antibody magnetic beads. It was shown that the NK cells concentration is a critical parameter during the optimization, as only at high concentration NK cells were able to promote tumor cell death before infiltration [65]. To improve the model relevance, Schnalzger et *al*. optimized a co-culture of tumor colon PDOs with NK cells. During the co-culture optimization they found that unlike T cells, NK cells killing and infiltration was dependent of extracellular matrix (ECM) density used in organoid co-culture [52]. Recently, an ALI culture method described by Neal et *al*., using PDO from primary and metastatic tumors was shown to retain stromal myofibroblast as well as immune cells like T, B, NK cells and macrophages. With this method, the authors showed the feasibility and the potential application of ALI-cultured organoids for testing the efficacy of immune checkpoint blockade treatments comprising a large proportion of TME components derived from patient samples [66].

#### Enrichment with stromal cells

Aside from immune cells, the TME consists of a variety of stromal cells. The stroma is defined as the surrounding ECM and the mesenchymal cells within it including endothelial cells (ECs) and cancer-associated fibroblasts (CAFs). Crosstalk between stromal, immune and cancer cells play a pivotal role in tumorigenesis and in treatment resistance in CRC [67].

ECs are the main components of the tumor vasculature, with angiogenesis being an important progression hallmark of cancer [68, 69]. Angiogenesis and TECs play critical role in CRC metastasis promoted by the development of neo-vascularization regulated by various pro-angiogenic factors (such as VEGF, PDGF, FGF, and angiopoietin) [70]. The angiogenic tumor vasculature, derived from activated ECs or stem cells, also expresses immunosuppressive molecules [71], that create a barrier for immune cells. The barrier function of the tumor vasculature is also caused by the lack of tumor endothelial cell adhesion molecules such as intercellular adhesion molecules (ICAMs), vascular cell adhesion molecules (VCAMs) and selectins - a phenomenon that is called endothelial cell anergy [72, 73]. This tumor endothelial cell anergy has been presented as a vascular immune checkpoint [74], that the tumor has hijacked from an embryonic regulatory mechanism [75].

Co-culturing organoids with ECs enhances the physiological relevance of the model by mimicking the tumor microenvironment more accurately. This approach enables the study of tumor-vascular interactions, angiogenesis, and drug responses in a more realistic setting. In a recent review, Strobel et *al*. presented various vascularization strategies within organoids, see Fig. 2 [76]. Important points raised were the use of tissue-specific endothelial cells, the culture conditions with the use of ECM and specific growth factors to support 3D structure and cell differentiation [76–78]. In this context, the addition of a perfusion system help in the development a vascular network within the organoid compared to static culture [79–82]. Investigating how CRC cells interact with endothelial cells can uncover new targets for anti-angiogenic therapies and combination therapies perspectives. Truelsen et *al*. introduced “The Cancer Angiogenesis Co-Culture assay” as an in vitro functional assay to study the treatment response to anti-angiogenic agents in organoids [83]. In their model, endothelial cells (HUVECs) were cultured in co-culture with fibroblasts, where HUVECs formed tube-like structures within the layers of fibroblasts and ECM. Two days later the PDOs were added to the co-culture. The authors highlighted the strong potential of regorafenib and bevacizumab, inhibitors of VEGFR and VEGF, respectively to inhibit endothelial tube formation in experimental conditions with PDOs compared to the control, with no tumoroids and no treatment.

Moreover, CAFs are responsible for the secretion of various chemo- and cytokines to promote the progression of CRC and might alter the immune cell population in the TME [84]. The CAFs are representing fibroblast population located within or surrounding the tumor [85]. In CRC the CAFs are often associated with poor prognosis and therapy resistance [86, 87]. The ‘mesenchymal-like’ CMS4 characterized by a high content of CAFs [88], represent an important number of cases in CRC (23% of the cases) [12, 89]. Therefore enriching organoid environment with CAFs was conducted to investigate their role in the TME remodeling, promoting tumor growth, invasion, and resistance to treatment. Strating et *al*., showed that such co-culture resulted in elevated expression of ECM components, increased glycolysis, or hypoxia [90]. Additionally, CAFs induced a partial epithelial-to-mesenchymal transition (EMT) in a cancer cell population, promoting immunosuppression and ECM stiffening. The co-culture medium composition revealed an elevated level of the immunosuppressive factors, such as TGF-b1, VEGF-A and lactate, responsible of the T cell proliferation inhibition. In the study of Luo et *al*., the addition of patient-derived CAFs to the organoids in a 2:1 ratio (Fig. 2), resulted in an enhanced tumor growth in CRC PDOs compared to culturing PDOs without CAFs, suggesting that the secretion of paracrine growth factors stimulated tumor progression. Transcriptional analyses revealed potential restoration of crucial survival pathways and cancer-CAF interactions, also observed in patient tumor tissues. Furthermore, drug efficacy testing in CRC PDOs and co-cultures with CAFs demonstrated increased resistance levels, emphasizing the importance of incorporating CAFs when assessing drug responses in PDOs [91].

Human colorectal organoid on-chip models are an emerging innovative methods that integrate microfluidic technology to create a more physiologically relevant system by ensuring a proper irrigation, cyclic strain and fluid shear stress mimicking in vivo conditions for the living cells (Fig. 2) [92]. The dynamic environment facilitates longer-term experiment and the investigation of tumor progression, metastasis, and the efficacy and safety of combination therapies [93, 94]. For example, Strelez et *al*., managed to optimize a chip model with CRC tumors, including both CAFs and endothelial cells, to study the influence of the TME on early metastatic spread [93]. This model can also be used to recapitulate the multicomponent structure of human organs to study dynamic interaction of the TME with treatment. Using a microfluidic chip, Jenkins et *al*., were able to evaluate the response and resistance to PD-1 blockade, by profiling the secreted cytokines in multi-cellular PDOs [95]. The engineering behind organoid on-chip models is challenging and still need improvement for standardization of the method for robust reproducibility but it unlocks new avenues of research by using models that are closer to in vivo [96].

Collectively, these co-culture models represent a valuable tool for the identification of promising new therapeutics and underscore the importance of multicellular interactions in this process. Alongside, future studies should be performed to standardize co-culture settings to harmonized protocols and to better replicate the treatments strategies.

### Enriched organoid model to develop combined immunotherapeutic strategies

Organoids isolated from CRC have emerged as powerful prognostic models for immunotherapy screening in the past few years. In this field, the immune context of cancer has a significant impact on patient response to immunotherapy.

The CMS2 and CMS3 are poorly immunogenic with low immune cell infiltration, whereas the CMS4 is strongly enriched with immunosuppressive cell populations and stromal cells. Those CMS represent MSS tumor phenotype and are less likely to respond to immunotherapy. It was demonstrated that an immune infiltration in CRC by CD4 T cell, CD8 T cell, B cell, NK cell, tertiary lymphoid structure and macrophages represent a good prognostic value for patients [14, 61, 97–100]. On the opposite M2 macrophages characterized by an anti-inflammatory phenotypes are associated with a poor prognosis [61, 101, 102].

Therefore, an organoid co-cultures with stromal and immune cells were established to recapitulate better the heterogeneity and microenvironment of a patient tumor, as well as provide a promising platform for assessing immunotherapy response and guiding treatment selection in patients who are unlikely to respond to current first-line therapy [103]. Optimization of immunologically representative scalable functional assays represent an important step toward increasing the value of PDOs as a preclinical model for immunotherapy screening. Several new technologies have been developed by different groups [104–108], in which PDOs are used to identify tumor-reactive T cells, assess a patient’s potential response to immune checkpoint blockade, and identify neoantigens in individual tumors, to which cell therapies could be applied. Selected studies are discussed below.

#### CAR-T cell therapy

Strategies to co-culture tumor organoids with autologous T cells have demonstrated clinically significant treatment optimization, demonstrating the potential and versatility of PDOs and T cell co-culture systems for immunotherapy screening. Chimeric antigen receptor-T (CAR-T) therapies consist of genetically modified T cells able to specifically recognize tumor antigens and eradicate cancer cells. CAR-T therapies are established FDA-approved treatment for several blood cancers, such a B cell leukemia or lymphoma, but still remain a challenge for solid tumors [109, 110]. Lack of efficacy of CAR-T therapy may be caused by the barrier function through the lack of adhesiveness of the tumor vasculature described earlier. In this line of thought it might be advantages to design CAR-T therapies against the tumor vasculature, as this may solve the vascular barrier problem [111, 112]. Another problem with CAR-T therapy in patients with solid tumors is discontinuation of therapy caused by on-target off-tumor toxicities [113, 114]. Those often occurring toxicities support the use of PDOs for pre-clinical development to prevent and resolve them by finding antigen exclusively expressed in tumor cells [109, 115]. In clinical settings, poor response to CAR-T-based treatments is also caused, among others, by the TME composition in solid tumors, therefore there is a need for adequate co-culture models with preserved TME [116]. Moreover, due to limitations in treating solid tumors with CAR-T cells, an unmet need remains to discover new targets and optimize combination strategies.

**Fig. 3:**
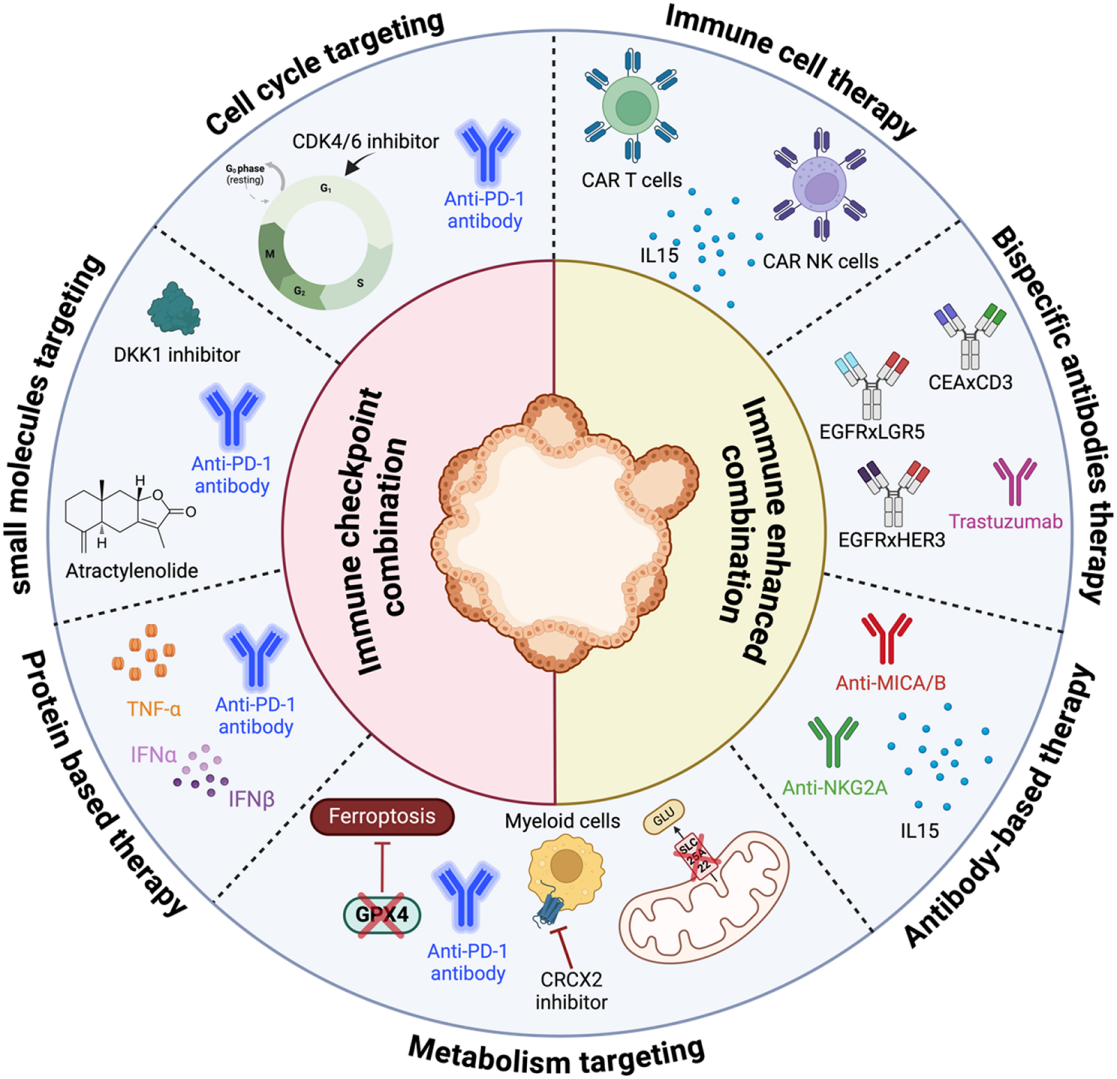
Schematic overview of the different immunotherapeutic combination strategies developed using PDOs. Created with BioRender

Schnalzger et *al*. proposed an original in vitro platform designed for pre-clinical testing of CAR NK cells using CRC PDOs as shown in Fig. 3 [52]. The authors demonstrated the response of CAR-NK cells to two clinically relevant cancer-associated antigens, namely epidermal growth factor receptor variant III (EGFRvIII) and the WNT receptor FRIZZLED (FZD). EGFRvIII-specific CAR-NK cells showed high selectivity for PDOs expressing mutant EGFR, with no off-target toxicity observed in healthy colon organoids. Conversely, FZD-specific CAR-NK cells showed non-specific killing of PDOs regardless of their origin (normal human colon organoids and primary fibroblasts derived from non-pathological mucosa collected either during preemptive colonoscopy or from tumor-adjacent normal colon after tumor resection) and FZD receptor status, suggesting that clinical targeting of FZD may lead to mucosal toxicity.

In CRC the expression of CD70 on CAFs has been identified as a novel negative prognostic marker and thus independently of the MMR status [117, 118]. The CD70 expression has been suggested to help tumor immune evasion and accelerate tumor growth. Of note, a higher CD70 expression by CAFs compared to tumor cells was showed in CRC cases (14,9% vs. 2,2%) [118]. Recently, Van den Eynde et *al*. have demonstrated that CD70 in CRC and pancreatic ductal adenocarcinoma patients could be a potential therapeutic target for both tumor cells and tumor promoting CAFs [119]. Using PDOs co-cultured with CAFs they showed that CD70-CAR-NK cells in combination with IL-15 is needed for an effective eradication of low- and high-expressing CD70 + tumor cells and CAFs.

The simultaneous administration of CAR-NK cells and CAR-T cells, could also increase the effectiveness of treatment, therefore the use of PDO can lead to a rapid identification CAR T/NK new target and test of their efficacy [120].

#### Immune checkpoint inhibitors (ICIs)

The use of immune checkpoint blockers such as PD-1/PD-L1 inhibitors, has emerged as a cornerstone in cancer treatment. The PD1 expression has been mainly described on cell surface of activated lymphocytes populations and particularly CD8^+^ T cell [121–123]. Recently, PD1 expression has also been described in a variety of immune cells (DCs, macrophages, NK cells) and tumor cells [124, 125]. The interaction of PD1 with its ligand PD-L1 impair T cell activation, cytotoxicity and induce an immune tolerance favorable for tumor cell onset [126, 127].

In 2017, FDA approved pembrolizumab and nivolumab, anti-PD1 monoclonal antibodies (mAb), for the treatment of adult and pediatric patients unresectable or metastatic CRC with mismatch repair deficient and microsatellite instability high (dMMR/MSI-H) that has progressed prior a fluoropyrimidine, oxaliplatin, and irinotecan treatment [128, 129]. In 2020, pembrolizumab has been approved as a first-line treatment for patients with unresectable or metastatic MSI-H or dMMR colorectal cancer [130]. However, its efficacy remains limited to patients with dMMR/MSI-H tumors, which represents approximately 15% of CRC patients [131]. Enhancing the efficacy of anti-PD1 therapy through different pathways is pivotal, driving significant research efforts towards identifying novel combination strategies (Fig. 3).

One of the strategies used by Deng et *al*. was to combine small molecules with immune checkpoint blockade (Fig. 3). Using MC38 murine-derived organotypic tumor spheroids, the authors demonstrated that a combination of CDK4/6 inhibitor (trilaciclib or palbociclib) with anti-PD1 achieve a greater killing effect. They further validated the combination efficacy in vivo in both partially anti-PD1 responder mouse model (MC38) and anti-PD1 resistant mouse model (CT26) [132].

A study by Sui et *al*. highlighted that an elevated Dickkopf 1 (DKK1) expression and an elevated concentration in serum was associated with recurrence, decreased CD8^+^ T-cell infiltration and poor response to PD-1 blockade in dMMR CRCs patients [133]. In their CRC organoid model, they validated the potential of DKK1 inhibition in combination with anti-PD1 with a further increase of apoptotic cells proportion when both were combined. Another small molecule-based drug Atractylenolide I, was identified through an CRC organoid model that showed promising MHC-I-mediated antigen presentation on CRC cells inducing a T cell infiltration and cytotoxicity which was strengthen the efficacy of anti-PD-1 in combination [134]. These studies extend the possible choice of therapeutic combinations for patient management. In these cases, organoid models are used for validation, as opposed to screening, which could yield a broader pool of new combinations.

A clinical study involving patients with early-stage CRC receiving neoadjuvant ICI have been used to evaluate the correlations between PDO-specific T cell reactivity and clinical responses to immunotherapy [135]. Most PDOs were generated from pre-treatment biopsy samples. However, for three clinical non-responders, post-treatment resection specimens were used as a source material. PDOs and clinical responses were only partially correlated, with reactivity seen for only three of six responders. One of the limitations in this study was that patients received the combination of an anti-PD-1 antibody and an anti-CTLA4 antibody, whereas only an anti-PD-1 antibody was included in the PDOs treatment. An alternative explanation for this lack of correlation might also be the absence of other important TME components. The only presence of reactive T cell doesn’t mimic the full spectrum of CMS, additional immune population like DCs or NK cells that are associated with a good response or Treg, MDSCs that are associated with poor response to immunotherapy can bring more valuable and relevant results [15, 136, 137].

For instance, in an enrichment model of HT29 spheroid co-cultured with T and NK cells from from healthy donors PBMCs, Coureau et *al*. showed that stimulating TILs and NK cells infiltrate induced an immune response capable of destroying tumor organoid structures. In their study they inhibited NKG2A a powerful inhibitory signal in both T and NK cells and the major histocompatibility complex-class I chain related proteins A and B (MICA/B) to avoid shedding on cancer cells and escape from NKG2D recognition by NK cells [138]. Their results highlight the powerful anti-tumor potential of IL-15-based treatments combined with immune modulatory anti-MICA/B and anti-NKG2A (Fig. 3) for cancer treatment for which co-cultured spheroids deeply help to characterize the efficacy and mode of action [139]. Although this study was performed in a spheroid model, similar experiments could very well be performed in this more complex model. With the enrichment of the PDOs model with myeloid-derived suppressor cells (MDSCs), Chen et *al*. showcased a critical role of those tumor-infiltrating MDSCs in resistance to PD-1 blockade in CRC. The authors demonstrated that a co-culture of CRC organoids expressing PD-L1 in the presence of MDSCs did not respond to anti-PD-1 therapy. However, when combining a simultaneous T cell activation and a depletion of MDSCs by IFN-α/β and TNF-α treatment (Fig. 3) in co-culture conditions, this led to an increased immunogenic organoids cell death following anti-PD-1 therapy [140].

In the last few years, the implication of myeloid compartment on immunotherapies response have been highlighted in several cancer types [137, 141–144], including CRC. This large population of myeloid cells are now considered as a clinical predictive biomarkers for ICIs [144]. Emerging strategies include combinatorial approaches with myeloid-targeting agents to improve ICIs response. A wide range of approaches is used to target this subsets, by either depleting them, preventing their recruitment, their differentiation and by inhibiting or reprogramming their immunosuppressive functions [142, 145]. However, their implication in primary or acquired resistance as well as the reciprocal interactions between tumor cells, dendritic cells or lymphocytes is still under investigation. For this purpose, co-culture of these cells in a complex 3D environment will provide further additional insight on the orchestration of their interactions and the resulting biological effect.

#### Other immune therapy-based combinations

Bispecific antibodies (BsAb) result of constant advances in engineering antibody field. The main benefit of BsAb is the dual targeting capability and the large number of combinations that can be generated [146]. The main benefit of BsAb is the dual targeting capability and the large number of combinations that can be generated [146]. A functional screening of more than 500 BsAbs on CRC PDOs has been done by Herpers et *al*., from the screening they identified MCLA-158 BsAb targeting EGFRxLGR5 that target highly mitotic leucine-rich repeat-containing G-protein-coupled receptor 5-positive (LGR5+) CSC population supporting organoid growth found in primary and metastatic tumors. This BsAb EGFRxLGR5 (Fig. 3) showed a higher efficacy demonstrated by lower IC_50_ on tumor PDOs and minimal toxicity through a higher IC_50_ on adjacent healthy mucosal tissue PDOs than Cetuximab. As well in vivo in an PDX orthotopic CRC model the BsAb EGFRxLGR5 showed a greater reduction of tumor size and weight compared to control [147]. Around 70% of CRC cases harbor multiple genetic alteration of the EGFR signaling pathway implicated in the resistance of targeted therapy such as anti-EGFR [148]. The combination strategies appears crucial to overcome either primary and acquired anti-EGFR therapy resistance [149]. Rau et *al*. demonstrated by using a newly developed bispecific EGFRxHER3-targeting antibody together with trastuzumab (Fig. 3) the efficacy of multiple targeting by CellTiterGlo^®^ assays on two different CRC cell line spheroids and PDOs [150]. These two studies highlighted that a better understanding of resistance mechanism combined with computational engineering approaches, could therefore facilitate the future of combined drug development.

A new immunotherapy known as CEA-TCB, an IgG-based T-cell bispecific antibody (TCB) reroutes T cells to tumor cells that express the carcinoembryonic antigen (CEA) glycoprotein on their cell surface, regardless of the T cell receptor specificity of the redirected T cells. Cibisatamab contains two antigen-recognition sites: one for human CD3, a T-cell surface antigen, and one for human CEA, a tumor-associated antigen that is specifically expressed on certain tumor cells [151]. Gonzalez-Exposito et *al.* showed that the heterogeneity of CEA expression contributed to resistance to cibisatamab in the T cell and CRC PDOs co-culture systems [152]. By exploring the pathways regulating CEA expression the authors found that a combination of cibisatamab and the WNT/β-catenin inhibitors (porcupine inhibitor LGK-974 and tankyrase inhibitor compound 21) (Fig. 3) can increase the drug sensitivity to cibisatamab in PDOs established with metastatic chemotherapy-resistant CRC patient samples. Thus, it became clear that this co-culture model might be used for the new prognostic biomarkers, as well as an approach to improve immunotherapy sensitivity in clinical settings.

#### Targeting tumor metabolism to promote immunity

Another recognized hallmark of cancer that gain attention over the years is the tumor metabolism [68, 153]. Deregulation of metabolism has been shown to promote tumor progression [154], influence the tumor immune microenvironment [155] and induce drug resistance [156, 157]. Targeting metabolic pathways have shown to promote immunity by enhancing anti-tumor T cells, reducing immunosuppressive populations and thus enhance immunotherapy response [158].

One of the metabolic pathways investigated by Conche et *al*. is the ferroptosis, i.e.an iron-dependent cell death, which cancer cells can bypass [159, 160]. They used a specific CRC organoids model harboring *ACP*,* Trp53*,* Tgfbr2*,* K-ras*^*G12D*^*(APTK)* mutations, similarly to the human mesenchymal CRC subtype (CMS4). The authors transplanted those organoids in immunocompetent mice. In vivo, the combination of ferroptosis induction by Glutathione peroxidase 4 inhibition (Withaferin A) along with PD-1 and CXCR2 inhibitor (SB225002) a MDSC blocker effective for CRC liver metastasis inhibition (Fig. 3), whereas primary CRC tumor was resistant to this combinatorial treatment [161]. This study suggests that PDOs can be used to identify an additional combination therapy effective for advanced mCRC. Furthermore, the authors demonstrated that targeting tumor metabolism influences T cell activation and cytotoxicity toward liver metastasis.

In the CMS3 subtype of CRC, the presence of *KRAS*-mutant is associated with low immune infiltration [12, 13, 15]. Emerging evidence describes the glutaminolysis process promoted by *KRAS*-mutant CRC generating critical metabolites and epigenetic deregulation to support cancer cell proliferation, stemness and chemotherapy resistance [162]. Zhou et *al*. identified SLC25A22 a mediator implicated in the glutaminolysis process. They took benefit of CRC organoids with *APC-KRAS* mutations and colon-specific *Slc25a22* knockout to inject them in immunocompetent mice. Similarly, they observed a reverse *KRAS*-mediated immunosuppressive phenotype hampering MDMCs recruitment, increasing cytotoxic T cell activation and finally synergy with anti-PD1 therapy [163].

**Table 1:** Immunotherapeutic combination strategies in CRC organoid model

Drug Combination	Model Used	Results	Reference
CDK4/6 inhibitor (trilaciclib or palbociclib) + anti-PD1	Patient and murine-derived organotypic tumor spheroids	Greater killing effect, validated in partially anti-PD1 responder (MC38) and anti-PD1 resistant (CT26) mouse models	Deng et al. [132]
DKK1 inhibitor + anti-PD1	CRC PDOs model	Increased apoptotic cell proportion and improved response to anti-PD1	Sui et al. [133]
Atractylenolide I + anti-PD1	CRC PDOs model	Enhanced MHC-I-mediated antigen presentation, T cell infiltration, and cytotoxicity, strengthening anti-PD-1 efficacy	Xu et al. [134]
anti-MICA/B + anti-NKG2A + IL15	CRC tumor spheroids with T and NK cells	Anti-MICA/B and anti-NKG2A was synergistic and enhanced immune-dependent destruction of tumor spheroid.	Coureau et al. [139]
IFN-α/β and TNF-α + anti-PD1	CRC PDOs model with MDSCs	Increased immunogenic organoids cell death following anti-PD-1 therapy	Chen et al. [140]
CD70-CAR-NK cells + IL-15	PDOs co-cultured with CAFs	Effective eradication of low- and high-expressing CD70 + tumor cells and CAFs	Van den Eynde et al. [119]
Ferroptosis induction (Withaferin A) + PD-1 inhibitor + CXCR2 inhibitor (SB225002)	CRC organoids in immunocompetent mice	Effective for CRC liver metastasis inhibition, primary CRC tumor resistant	Conche et al. [161]
EGFRxLGR5 bispecific antibody (MCLA-158)	CRC PDOs and PDX orthotopic CRC model	Lower IC50 on tumor PDOs, minimal toxicity on adjacent healthy mucosal tissue PDOs, greater tumor reduction in vivo	Herpers et al. [147]
Bispecific EGFRxHER3-targeting antibody + trastuzumab	CRC cell line spheroids and PDOs	Efficacy demonstrated by CellTiterGlo assays on different CRC models	Rau et al. [150]
Cibisatamab + WNT/β-catenin inhibitors (LGK-974 and compound 21)	T cell and CRC PDOs co-culture systems	Increased drug sensitivity to cibisatamab, overcoming resistance due to CEA heterogeneity	Gonzalez-Exposito et al. [152]
SLC25A22 knockout + anti-PD1	CRC organoids with APC-KRAS mutations in immunocompetent mice	Reversed KRAS-mediated immunosuppressive phenotype, enhanced T cell activation, and synergy with anti-PD1 therapy	Zhou et al. [163]

As listed in Table 1 drug development and screening using immune co-culture models of PDOs are emerging bringing a wide range of new combination strategies. However limitations remain, the current co-culture models of PDOs fail to fully reproduce the original TME as in vivo system, limiting their application to target accessory immune and microenvironment cells. Some studies lack immune cell components cultured together in PDOs, others use non-autologous immune cells which could lead to unreliable prediction. Clinical application of combined immunotherapeutic regimen needs extensive trials and higher number of patient samples due to individual differences and the distinct roles of various immune cells in the immune system. Furthermore, toxicity of the novel combined therapies is not consistently addressed which could provide valuable insight to identify off-target effects and assess the safety profile for future clinical application.

### Challenges and future perspectives

In the chapters above, we discussed advancements in the use of human patient-derived organoids in new combinatory treatments discovery for CRC. Even though organoids are more and more available in academic and industrial institutions, their use for the identification of combination strategies is relatively low. This can be explained by several factors.

Firstly, there is the scientific culture, which frequently translates results from drug candidates tested in simple 2D in vitro models directly to in vivo animal models. To implement PDOs model tests in predictive/drug discovery clinical standard of care, it necessitates neighboring clinical infrastructure, established collaborative network between scientists and clinical partners, such as surgeons, clinical pathologists or clinical oncologists as well as necessary ethical agreements. To be introduced in a clinical routine in addition to the tumor molecular profiling, PDOs generation need to be achieved in a clinical timeframe for patient care. CRC studies report a timeframe from around 2 weeks up to 53 weeks [19, 21, 164, 165] with a median time of 9 weeks [164] for the entire process. Standardization and automatization could reduce the timeline and variability for PDOs establishment and drug selection for patient benefit.

Another, more technical points are the standardized and reproducible protocols for maintenance, culturing/seeding, cryopreservation and treatment of organoids, especially those enriched with various cell types [166]. Not only organoids are known to be heterogenous between the patients, but the addition of other cells type requires an optimized cell culture medium to maintain the cells alive and enable their proliferation. The protocols should include human factor, i.e. possible sources of errors, experience, lab-to-lab equipment differences, that all may greatly influence the result of experiments. Quality control plan should be available with each validated protocol to secure the most reproducible and reliable outcome. In addition, the use of organoids technology in high-throughput drug-screening requires a standardized step of seeding, for the size of the organoids should be clinically relevant in order to obtain relevant and reproducible results [19]. Standardization of in vitro drug screening assays in organoids model is also to consider. There are multiple assays based on luminescence, colorimetry or fluorescence to assess the cell viability in PDOs. Even if the most used readout in the literature is the CellTiter-Glo^®^, that reflects cell viability based on the ATP quantification, other tests related to cytotoxicity are also used and should be considered or standardized using a decision tree based on the PDOs model and culture settings. Optimization of medium during co-culture appears essential for all cell types maintenance [167], as shown by Neal et *al*., by IL-2 addition for immune components maintenance viability [108]. The use of automated platforms in cell culture has widely emerged and allowed to perform high-throughput screenings in less time and effort. However, it comes with different type of challenges such as inconsistent volume measurements due to various viscosities of the solutions used, sample contamination, and even damage can result from poor pipetting techniques. This could lead to an accumulation of errors, inadequate data, poor reproducibility, and enhanced costs [168, 169].

As seen in the studies discussed above, only a few of them [19, 35, 52, 147] use simultaneously non-cancerous organoids (colon organoids) to evaluate safety of drug candidates. This could very well be due to the fact that the isolation and maintenance of colon organoids requires different conditions than CRC organoids, therefore makes the entire process even more complicated. It should be underlined however that the efficacy of combination therapy may come along with more important side effects [170]. Therefore, mathematical models are being developed alongside screening tests to evaluate drug-drug interactions, as well as supporting data for dose and drug selection to achieve synergistic combinations [19, 170, 171]. The recent development of multi-organ organoids is particularly useful in studying drug-induced liver, heart, kidney, gastrointestinal, and brain toxicities [172–175]. Ongoing and future advancements in organoid-based technology has the potential to provide more accurate and reliable models for drug safety profiling, ultimately leading to safer and more effective therapeutic strategies​.

Optimization of combinatory approaches containing 3 or 4 drugs based on drug synergy remains rather rare. This is a consequence of the current development of methods for multi-drug combinations and the assessment of their interactions. It is known that anti-cancer drugs as single agents have high attrition rate, where only 5% of the drug-candidates make it to Phase III clinical trials [176–178]. Surprisingly, this rate has not improved in a decade despite the major advances in experimental research [179]. This might be due to the lack of relevant pre-clinical models that would recapitulate the complex and heterogenous nature of the human physiology. When it comes to drug combinations, the issue is not simpler, even though most drug candidates consisting of these drug mixtures are already used in the clinics. This is mainly due to a drug-drug interaction. This parameter is not always evaluated and depends highly on the complexity of the model used and changes from a model to another (i.e. PDOs vs. PDOs enriched with immune cells). This leads to a more challenging evaluation of the activity and toxicity of a drug combination, and therefore a more difficult translation from in vitro to in vivo. Other challenges for immunotherapy-based combinations are being studied as are the immune CAR-T or NK cell generation, constantly under development to reduce the production timeframe and improve their stability overtime, as well as the high cost of manufacturing that restrains their use in the clinics [180].

Cancer organoids do not present the complexity of living organism limiting the tests of several therapies, e.g. anti-angiogenic therapies or those that require in vivo metabolization [181, 182]. A crucial tumoral parameter is its heterogeneity in terms of phenotype, genotype or cell composition, which underscores the importance of using combination therapies. Building a complex co-cultured organoids model is a way to fill the gap between the primary tissue [108]. New technologies such as spatial transcriptomics and multiplex imaging have highlighted the significant impact of exploring the crosstalk between stromal, immune and cancer cells [183–186]. For example, Li et *al*. by mapping cell distribution of CRC patient samples, mouse bearing CRC tumor and co-culture model of CRC PDOs revealed the advantage of PDOs to investigate the functional interactions between CRC and TME [187]. Intricate interaction between ECs and immune cells have been described in tumor [188, 189], the investigation of such interactions in PDOs could enhance therapeutic strategies for CRC patients. The use of complex PDOs therefore could provide dynamic insight revealing the optimal drug administration regimen and the cells crosstalk in real-time leading to drug response or resistance.

Another aspect developed recently is the mimicking of the organ architecture. A mini-intestine organoid model developed by Lutolf et *al*., can conserve intestinal characteristics and survive couple of weeks with microfluidics perfusion [190]. This model could allow even more relevant and physiological interactions between various cells. Another study reports a successful addition of immune cells and microbiome into an Intestine-on-Chip model [191]. The combination of both chips would be an accurate representation of the in vivo situation to further study CRC without the necessity of actual in vivo models and compatible with high-throughput screening. Challenges related to the implementation of organoids in CRC research are listed in Table 2.

Finally, PDOs co-culture with various cell types, combined with multi-omics analysis, could provide comprehensive insights into tumor biology, heterogeneity, and therapeutic responses [90, 192]. The results can enhance the ability to identify novel pharmacological targets, diagnostic biomarkers as well as further investigation of mechanism of action or resistance for a better clinical translation [193].

**Table 2:** Challenges related to the implementation of organoids in colorectal cancer research

Challenge	Details	References
Standardisation & Automation	- Clinical timeframe for PDOs generation variability. - Lack of standardized and reproducible protocols for maintenance, culturing, cryopreservation. - Maintaining viable co-culture conditions for various cell types, including the need for optimized culture media.	[19, 166–169]
Drug Screening	- Standardization of seeding density and organoid size for drug screening assays. - Inter- and intra-experimental variability in high-throughput screening. - No defined benchmark tests to assess drug efficacy/toxicity.	[19, 108, 168, 169]
Multi-Drug Combinations	- Drug-drug interactions and model variability. - Difficult evaluation of drug activity and toxicity. - Necessity to develop mathematical models to evaluate drug-drug interactions, dose selection and synergistic combinations.	[19, 176–179]
Complexity of Organoids	- Organoids lack the complexity of living organisms. - Heterogeneity of tumors in phenotype, genotype, or cell composition complicates the use of combination therapies. - Immunotherapies to be tested require more complex co-cultured model.	[108, 181, 183–189]
Mimicking Organ Architecture	- Mimicking organ architecture that conserves tissue specific characteristics. - Lack of models that allow accurate in vivo situation like cell-to-cell interaction, migration.	[190, 191]

## Conclusion

CRC organoids are one of the earliest and well established pre-clinical models that has been widely used in translational research. Crucial research findings are consistently arising, and these organoid systems offer promising avenues for application. The ability to detect non-responders would save patients from the adverse side effects of an inadequate therapy, and PDOs from non-responders may benefit of a drug screening pipeline to uncover novel drug combination regimens. Challenges remain in the reproducibility and consistency of organoid culture, the lack of standardized processes, and the incomplete replication of the tumor microenvironment. Further validation and refinement of organoid-based approaches are warranted to maximize their clinical utility and impact on improving outcomes for CRC patients.

## Data Availability

All data generated or analysed during this study are included in this published article.

## Abbreviations

2D: Two-dimensional
3D: Three-dimensional
ALI: Air-liquid interface
CAFs: Cancer-associated fibroblasts
CAM: Cell adhesion molecules
CAR: T-Chimeric antigen receptor-T
CEA: Carcinoembryonic antigen
cDC2s: Conventional DC type 2
CK: Cytokeratin
CMS: Consensus molecular subtype
CRC: Colorectal cancer
CRIS: A-CRC intrinsic subtype A
DCs: Dendritic cells
DKK1: Dickkopf 1
ECs: Endothelial cells
EGFRvIII: Epidermal growth factor receptor variant III
EMT: Epithelial-to-mesenchymal transition
FZD: FRIZZLED
GM: CSF-Granulocyte-macrophage colony-stimulating factor
HUVEC: Human umbilical vein endothelial cells
ICIs: Immune Checkpoint inhibitors
ICAMs: Intercellular adhesion molecules
IPSC: Induced pluripotent stem cells
LGR5+: Leucine-rich repeat-containing G-protein-coupled receptor 5-positive
M-CSF: Macrophage colony stimulating factor
mCRC: Metastatic colorectal cancer
MDSCs: Myeloid-derived suppressor cells
MICA/B: Major histocompatibility complex-class I chain related proteins A and B
MMR: Mismatch repair
MSI-h: Microsatellite instability-high
MoDCs: Monocyte-derived dendritic cells
MMS: Microsatellite stablility
NK cell: Natural Killer cell
ODC: Optimized drug combination
PBMCs: Peripheral blood mononuclear cells
SIRT1: Sirtuin 1
TAM: Tumor-associated macrophages
TCB: T-cell bispecific antibody
PDOs: Patient-derived organoids
TECs: Tumor endothelial cells
TGMO: Therapeutically guided multidrug optimization
TME: Tumor microenvironment
VCAMs: Vascular cell adhesion molecules
